# Sport-Specific Assessment for Change of Direction Ability in Taekwondo: A Brief Review with Guidelines for Testing

**DOI:** 10.3390/sports14100425

**Published:** 2026-10-01

**Authors:** Gennaro Apollaro, Chris Bishop, Rafael L. Kons, Emerson Franchini, Daniele Detanico, Piero Ruggeri, Coral Falcó, Emanuela Faelli

**Affiliations:** 1 Department of Neuroscience, Rehabilitation, Ophthalmology, Genetics and Maternal Child Health, University of Genoa, 16132 Genoa, Italy; gennaro.apollaro@edu.unige.it; 2 Centro Polifunzionale di Scienze Motorie, University of Genoa, 16132 Genoa, Italy; ruggeri@unige.it (P.R.); emanuela.faelli@unige.it (E.F.); 3 Faculty of Science and Technology, London Sport Institute, Middlesex University, London NW4 4BT, UK; c.bishop@mdx.ac.uk; 4 Department of Physical Education, Federal University of Bahia, Salvador 40170-110, Brazil; rafael.kons@ufba.br; 5 Martial Arts and Combat Sports Research Group, Sport Department, School of Physical Education and Sport, University of São Paulo, São Paulo 05508-030, Brazil; efranchini@usp.br; 6 Biomechanics Laboratory, Center of Sports, Federal University of Santa Catarina, Florianópolis 88040-900, Brazil; d.detanico@ufsc.br; 7 Department of Experimental Medicine, Section of Human Physiology, University of Genoa, 16132 Genoa, Italy; 8 Department of Sport, Food and Natural Sciences, Western Norway University of Applied Sciences, 5020 Bergen, Norway

**Keywords:** combat sports, performance, Olympic sports, physical tests, agility training

## Abstract

In taekwondo combat, agility is essential for rapid movement and maintaining match pace, as well as for executing unilateral technical actions swiftly in multidirectional planes. Furthermore, it is also required to dodge and block opponents’ repeated attacks and to handle clinch situations and close-quarters combat effectively. This brief review aims to present the sport-specific tests available in the current literature for change of direction (COD) assessments in taekwondo, summarizing and analyzing the development and validation process of each test. This narrative review was conducted in accordance with the SANRA checklist. A literature search of PubMed/MEDLINE, Google Scholar and Scopus was performed for research articles from September 2017 to February 2026. The inclusion criteria were studies focusing on taekwondo athletes of any competitive level, age category, and nationality; evaluating COD through sport-specific testing; reporting at least one aspect of the validity, reliability, or sensitivity of the test; or providing a practical application of the test protocol. A total of 113 records were identified across databases. After deduplication, screening, and full-text assessment, 26 articles focused on the specific assessment of COD in taekwondo using three currently available tests (i.e., the Taekwondo-Specific Agility Test, the Taekwondo-Specific Aerobic–Anaerobic-Agility test, and the Taekwondo-Specific Change-of-Direction Speed Test) were included in the results. These tests incorporate greater sport-specificity and may provide greater ecological relevance than general tests, as they mimic the typical movements and technical actions of the match. However, the distinct methodological and measurement characteristics of each test must be considered in practical use. Future research should explore further aspects of validity (such as face, convergent and predictive validity), as well as clarify the reliability (i.e., test–retest and/or intra-/inter-rater) and sensitivity of these tests and develop classification tables. Practitioners can use the available tests to monitor the acute and chronic effects of training protocols on sport-specific COD performance, to monitor the relationship between the latter and other fundamental fitness components, or to classify athletes.

## 1. Introduction

The journey that will lead taekwondo to the 2028 Los Angeles Olympic Games (OG) has begun with significant changes to the rules of competition, designed to make them easier for the public to understand and to ensure greater fairness and consistency in scoring [1]. Among the main changes, valid spinning kicks that strike the electronic headguard will be awarded six points, representing an increase of one point compared with the previous ruleset [1,2]. Valid spinning kicks to the electronic body protector continue to award the same score (four points), as do valid linear and circular kicks to the electronic body and headguard protectors (two and three points, respectively) [1,2]. Furthermore, coaches can no longer request instant video replay for kicks to the head, to ensure that only hits that produce a clear impact are counted [1,2]. The latest technological development involves also extending the electronic scoring system to punches (which continue to be awarded one point) through a semi-automatic assessment in which the system measures impact and the judges assess the accuracy [1,2]. In this context, Olympic- and other-level (i.e., international, national or regional) taekwondo competitions are regularly reassessed, as each Olympic four-year cycle is characterized by specific rule changes and innovations that affect the high level of skill and technical–tactical variety [3,4,5], as well as the physiological demands of the sport [6,7,8,9,10]. Physiological responses are generated by the technical–tactical interactions between the two opposing athletes within the octagonal area enclosed in a square, which establish a highly specific combat pace [11]. Additionally, to make taekwondo not only more dynamic, but also more appealing to the public, changes to the scoring rules and the introduction of the electronic scoring system led to an increase in the variety of technical and tactical actions used. This has led to the categorization of corrective kicks (i.e., kicks performed either with the same leg or the opposite leg, following a bottom-up or top-down trajectory), thereby emphasizing the unilateral nature of kicking techniques [4,5].

In this context, agility is essential for rapid movement during combat and maintaining the match pace imposed by the new rules [3,10], as well as for executing unilateral technical actions swiftly in multidirectional planes [12]. Agility is defined as a rapid whole-body movement with a change in velocity or direction in response to a stimulus, to the opponent’s actions, and to the opportunities and constraints of the environment [13,14,15,16,17]. During taekwondo combat, the stimulus that determines rapid movement is not limited to reaching the opponent’s body and headguard protectors in order to score points. In fact, agility is also required to dodge and block opponents’ repeated attacks, to handle clinch situations and close-quarters combat effectively, and to avoid penalties for stepping over the boundary line of the competition area. Therefore, agility is closely linked to the tactical, cognitive and psychological skills needed by an athlete [14]. These skills are developed over time, in which athletes refine their ability to scan the environment and identify potential patterns of combat based on what has happened previously in similar scenarios [15,16,17]. Once the relevant stimuli have been assessed, the athlete makes a decision and executes the response [17]. Thus, the stimulus and the resulting physical component are closely interlinked, which makes it difficult to assess agility and identify potential deficits [18]. However, when the change of direction (COD; i.e., the specific event of changing direction, which can occur during both planned conditions and agility conditions [19]) is examined separately, the assessment allows the identification of shortcomings in the physical component due to the task being pre-planned, therefore reducing the influence of any perceptual–cognitive demands [18,19,20]. COD incorporates key qualities associated with athletic performance such as acceleration, deceleration, and directional changes [19]. Furthermore, technique, straight sprinting speed, and leg muscle qualities are considered key factors underpinning COD ability [13].

### General Assessment of Change of Direction Ability in Taekwondo

Physical fitness tests are widely used in the sporting context as fundamental tools for tailoring training programs and objectives, for identifying athletes’ strengths and areas for improvement, and for the selection and development of talent [21,22]. Critically evaluating the literature published up to 2013 on the physical and physiological characteristics of taekwondo athletes, Bridge et al. [8] documented a significantly wider use of general tests (i.e., tests that do not reproduce the physical, physiological, technical, and tactical demands of a particular sport), both field- and laboratory-based, to assess endurance and anaerobic components compared with COD ability. Indeed, up to that point, only two studies had examined COD performance using general field-test protocols such as the Side Step Test and the 10 × 5-m shuttle run tests [23,24]; thus, this highlights the need for further research focused on this physical fitness component [8]. With this in mind, it is relevant to note that the subsequent literature has assessed COD ability in taekwondo athletes using various general field tests, such as the *t*-Test [25,26], modified *t*-Test [27,28], Illinois Agility Test [29,30], 10 × 5 m shuttle run test [31,32], and 5–0–5 Agility Test [33,34] (Table 1). In particular, the *t*-Test, which is one of the more commonly used assessments for pre-planned COD ability, revealed performance ranging from 9.8 ± 0.4 [35] to 12.5 ± 1.0 s [25] among regional- and national-level taekwondo athletes.

The use of these tests has provided useful evidence regarding COD performance in taekwondo athletes of different ages and competitive levels. However, the lack of mechanical specificity in these tests relative to typical movements performed during the low-intensity active phases of a match (e.g., hopping, shifts, and guard changes) and technical–tactical actions of taekwondo had already been recognized by Bridge et al. [8] as a factor limiting their validity and applicability. Accordingly, a growing body of research in taekwondo, and in Olympic combat sports more generally, has particularly focused on developing new and more specific tests (to assess different physical fitness components) that provide more ecologically valid alternatives to general assessments [45].

## 2. Sport-Specific Assessment of Change of Direction Ability in Taekwondo

Recent narrative reviews [6,7] have documented the availability of several specific testing protocols for taekwondo, with distinct methodological and measurement characteristics, to assess endurance and anaerobic components, better reproducing physical and physiological demands than general tests. The authors [6,7] justified the particular focus of the research on these two fitness components as a result of growing evidence regarding the role of energy systems, and the dynamics of the interaction between them, during competitive taekwondo matches [8,9]. A 2017 systematic review already found that 75% of the literature on sport-specific assessment in taekwondo focused on endurance and anaerobic components [45]. In contrast, COD ability was significantly under-represented and, up until that point, the Taekwondo-Specific Agility Test (TSAT) [12] was the only test available for assessing this fitness component in a sport-specific setting. In this context, considering the wide availability of sport-specific tests for other fitness components, the literature on the development, validation and use of sport-specific testing protocols for COD ability has seen an increase following the development of the TSAT.

## 3. Aim

This brief review aims to present the sport-specific tests available in the current literature for COD assessments in taekwondo, summarizing and analyzing the development and validation process of each test.

## 4. Methods

This brief review was conducted in accordance with the SANRA (Scale for the Assessment of Narrative Review Articles) checklist to ensure methodological transparency and rigor [46]. The protocol was retrospectively registered on the Open Science Framework (OSF; https://doi.org/10.17605/OSF.IO/3NF6U). PROSPERO does not accept narrative review protocols, so OSF was used for registration. The decision to conduct a narrative review was driven by the relatively recent development of the literature on the use of sport-specific testing protocols for COD ability in taekwondo compared to those for other fitness components. This has resulted in considerable heterogeneity in the available evidence in terms of sample characteristics, validity, reliability, and sensitivity, as well as practical applications. This diversity precludes the use of standardized methodological approaches required for systematic reviews or meta-analyses. Therefore, a narrative review was the most appropriate approach for synthesizing and discussing the results of currently available sport-specific tests of COD ability. Furthermore, this method aligns with the aim of providing recommendations, future perspectives, and practical applications as guidelines for scientists, coaches, and strength and conditioning professionals, based on a well-defined research strategy and the authors’ direct experience with the topic under investigation [6,7].

### 4.1. Literature Search

A literature search of PubMed/MEDLINE, Google Scholar and Scopus was performed for peer-reviewed original research articles, from September 2017 to February 2026, without restrictions regarding language. The search strategy combined the following descriptors: [(“taekwondo” OR “tae-kwon-do” OR “taekwon-do”) AND (“agility” OR “change of direction” OR “COD”) AND (“test” OR “fitness test” OR “assessment”)].

### 4.2. Eligibility Criteria

Inclusion criteria:

• Studies focused on taekwondo athletes of any competitive level, age category and nationality;
• Studies evaluating COD through sport-specific testing;
• Studies reporting at least one aspect of the validity, reliability, or sensitivity of the test, or providing a practical application of the test protocol.

Exclusion criteria:

• Studies that did not report primary empirical observations, such as review articles, editorials, and commentaries.

### 4.3. Additional Sources

The electronic search was supplemented by manual inspection of the reference lists of the retrieved articles to identify additional studies that matched the key search terms and theme of the review.

### 4.4. Study Selection and Screening Process

After the removal of duplicate records, titles and abstracts were screened for relevance, followed by full-text assessment of potentially eligible articles.

### 4.5. Quality Appraisal

This narrative review met all SANRA criteria [46]. The importance of the review was strongly justified based on the clear gap in current knowledge regarding sport-specific COD assessments in taekwondo (Score 2). The aims and research questions were well defined and explicitly formulated (Score 2). The literature search strategy was detailed, involved multiple databases, and followed transparent procedures (Score 2). Referencing was complete, current, and formatted according to guidelines (Score 2). Scientific reasoning was coherent and evidence-based, demonstrating clear synthesis across studies (Score 2). The presentation of data was appropriate, incorporating structured summaries, tables, and figures to support clarity and readability (Score 2).

### 4.6. Bias and Limitations

The potential biases and limitations inherent to narrative review are acknowledged in Section 6.4.

## 5. Results

**Figure 1:**
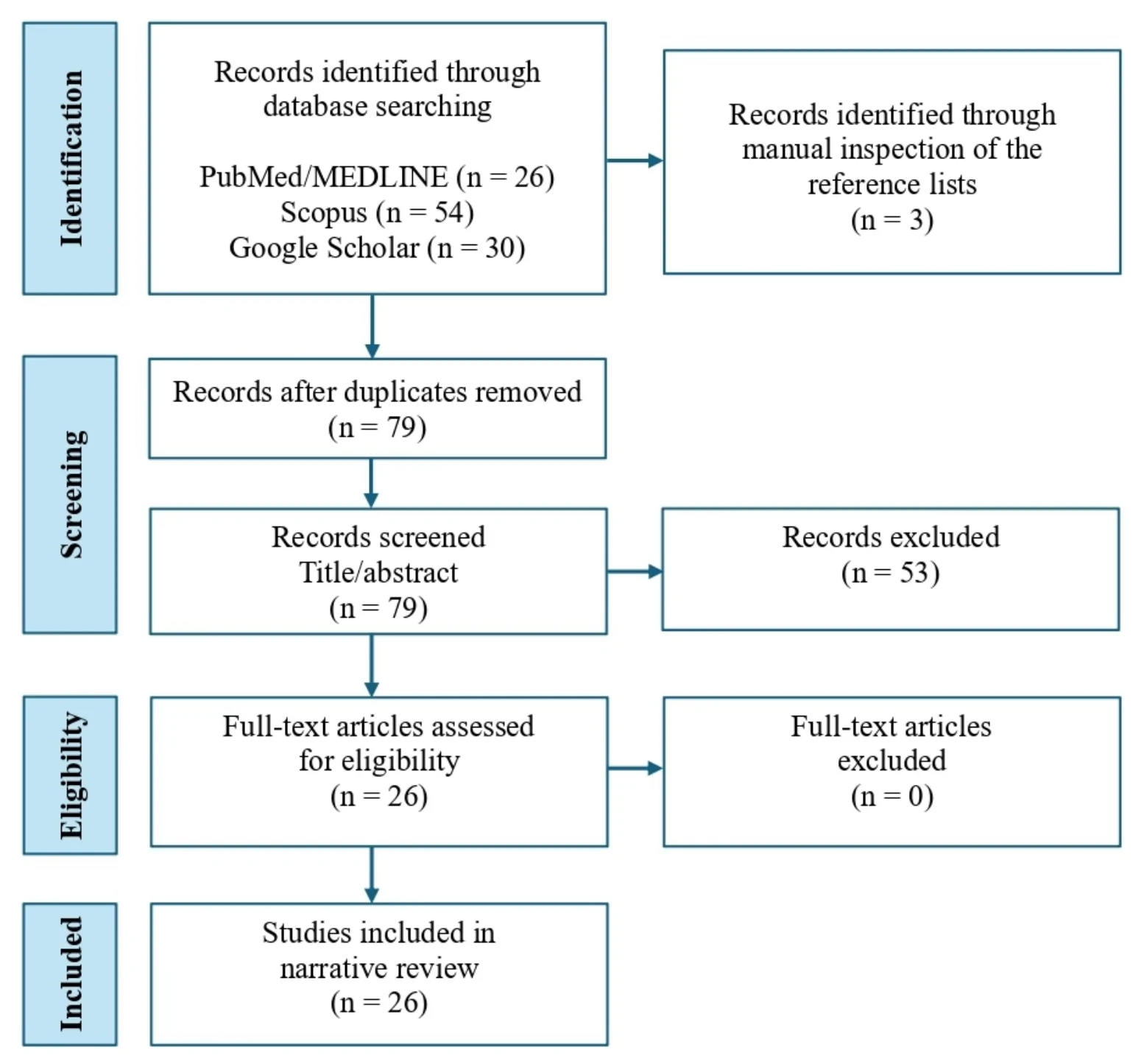
Flow diagram of the literature search and study selection process.

Based on the search criteria described, n = 26 articles focused on the sport-specific assessment of COD in taekwondo were identified. Specifically, three sport-specific tests were identified in the current literature for assessing COD in taekwondo: the TSAT (n = 24 articles) [12], the Taekwondo-Specific Aerobic–Anaerobic-Agility (TAAA) test (n = 1 article) [40], and the Taekwondo-Specific Change-of-Direction Speed Test (TST) (n = 1 article) [27]. The study selection process is summarized in a flow diagram (Figure 1).

The results from the studies in this review are outlined discursively below, summarizing and analyzing the development and validation process of each test.

### 5.1. Taekwondo-Specific Agility Test (TSAT)

**Figure 2:**
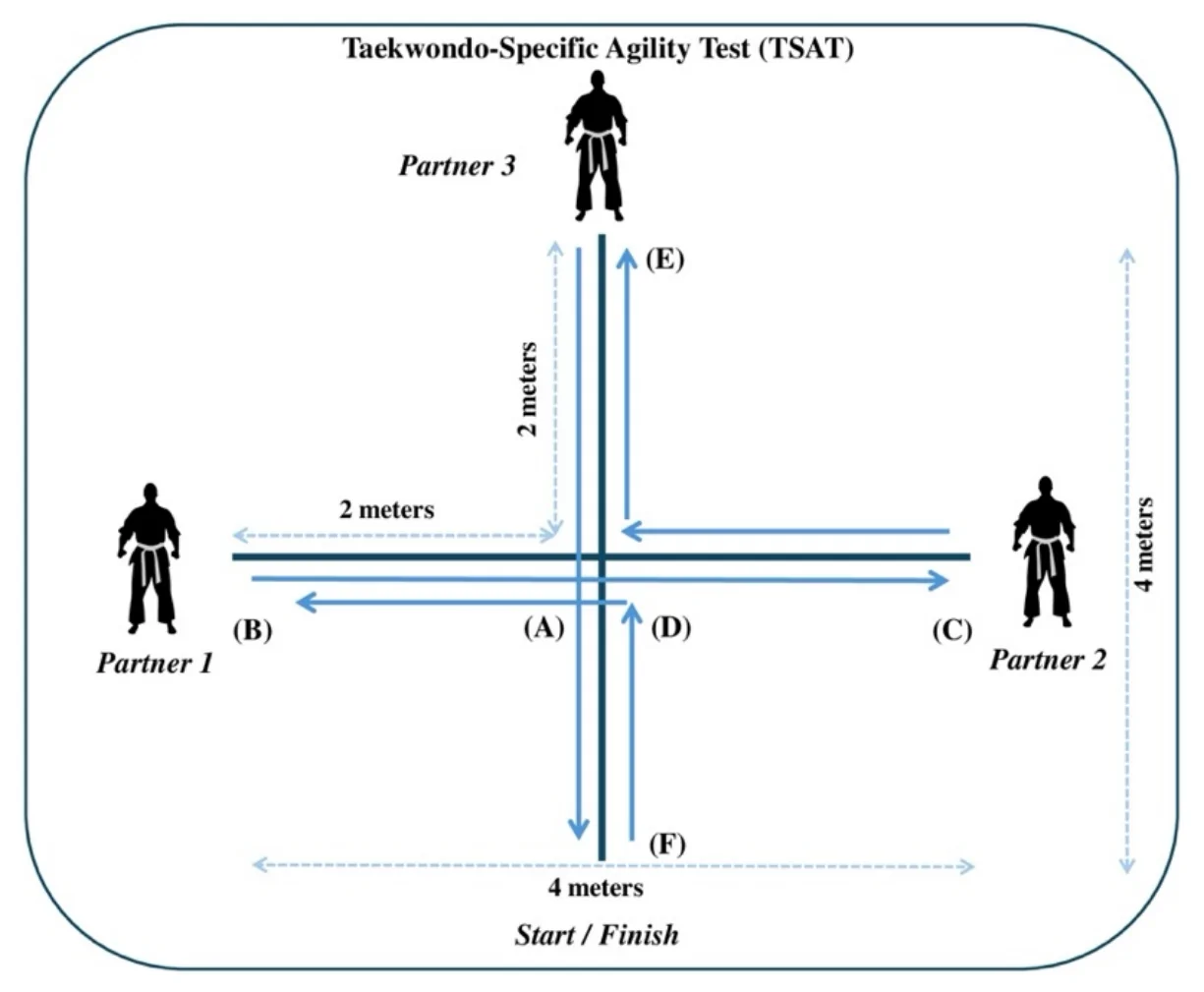
Taekwondo-Specific Agility Test (TSAT) [12]. From a guard position with both feet behind the start/finish line, the athlete has to: (A) move forward in guard position without crossing feet as quickly as possible to the center point; (B) turn toward partner 1 by adopting a lateral shift and perform a dollyo-chagi (i.e., roundhouse kick) with the left leg; (C) move toward partner 2 and perform a dollyo-chagi with the right leg; (D) return to the center; (E) move forward in guard position and perform a narae-chagi (i.e., double-roundhouse kick) toward partner 3; and (F) move backward to the start/finish line in a guard position. Partners 1 and 2 hold a striker, whereas partner 3 holds two strikers. They are instructed to maintain the striker at the torso height of the athlete. If the athlete crosses one foot in front of the other during the various displacements or fails to touch the target powerfully, the trial is invalidated and a new attempt is granted after a 3 min recovery period. The time to complete the test is used as performance and it is measured with an electronic timing system. The athlete performs three trials, and the best one is used for subsequent analysis.

Chaabene et al. [12] developed the TSAT, which consists of performing specific movements and three kicks (i.e., dollyo-chagi and narae-chagi), within a 4 m × 4 m area, with the assistance of three strikers supported by partners. Performance is determined by completing the test in the shortest possible time and with an overall technique deemed effective by the evaluator (Figure 2).

The authors determined certain aspects of the validity, reliability, and resulting sensitivity of the new test [12]. Specifically, they studied the concurrent criterion validity (i.e., the strength of the relationship and agreement between the sport-specific test and the established reference COD test [45]) of the TSAT with the *t*-Test. A very large and significant [47] correlation (Pearson’s *r* = 0.71) and a 50% shared variance between the performance of the two tests emerged [12]. In parallel, the discriminant construct validity of the TSAT (i.e., its ability to detect differences in the performance of taekwondo athletes of different abilities [45]) was investigated by comparing the performance between national- and international-level athletes. International athletes showed significantly (effect size [ES] = 2.2, “large”) superior COD performance (4.5 ± 0.4 s) compared to their national counterparts (5.3 ± 0.4 s). Furthermore, analysis of the receiver operating characteristic (ROC) curve revealed an area under the curve of 0.94 (with a cutoff value of ≤4.77 s for acquiring a high-performance level). An area under the ROC curve > 0.70 is commonly considered indicative of good discriminant validity [45]. Overall, these results indicated a high ability of the TSAT to discriminate between athletes of different competitive levels [12].

Regarding test–retest reliability analysis (i.e., the consistency of measurements or of a performance over repeated testing sessions [45]), the intraclass correlation coefficient (ICC; 0.97) and the coefficient of variation (CV; 1.8%) established an excellent relative and absolute reliability [47,48], respectively [12]. In this context, subsequent studies have systematically documented the test–retest reliability coefficients of the TSAT, particularly from a relative perspective. Specifically, the TSAT performance showed ICCs ranging from 0.72 to 0.98 [44,49] and a CV of 13.6% [44] (Table 2). In contrast, other studies have reported the results of relative and absolute intra-session reliability. They showed ICCs ranging from 0.80 to 0.90 [34,50,51] and CVs ranging from 9.3% to 10% [50,52], although the number of attempts allowed in these studies varied from two to three; thus, this somewhat limits the comparability of the results (Table 2). Overall, these subsequent studies reported a slightly elevated CV (i.e., above 5%) [48], which does not confirm the excellent absolute reliability observed in the initial study. In this regard, Chaabene et al. [45] suggested that the poor methodological quality of the studies, particularly regarding familiarization sessions, test–retest intervals, and the stability of testing conditions, may negatively affect the reliability of measurements and lead to subsequent issues when interpreting results. Specifically, familiarization was generally addressed in studies investigating the TSAT’s reliability to reduce measurement error associated with learning effects, although no standardized procedures were adopted or proposed. The test–retest interval depends on the test’s characteristics, including complexity, duration, and the type of effort required. In line with the literature [45], Chaabene et al. [12] applied a 7-day interval, whereas other subsequent studies did not report the exact time interval between trials. Moreover, other factors, such as environmental conditions and the fact that the details of testing conditions (and any variations) have not always been explicitly reported, may also influence subsequent test scores (and, therefore, measurement error). The sensitivity analysis revealed that the TSAT is a suitable test for detecting small but meaningful changes in performance, as its smallest worthwhile change (SWC_0.2_: 0.13 s; SWC_0.6_: 0.38 s) was higher than the typical error of measurement (TEM; 0.09 s). Meanwhile, the minimal detectable change (MDC_95%_) was 0.25 s, representing an estimation of the smallest change in score that can be objectively detected by the TSAT [45]. In light of this information, summary data for the TSAT assessment, including mean scores and reliability, are presented in Table 2.

The secondary aim of the study by Chaabene et al. [12] was to establish the relationship of the TSAT with lower-limb muscle power tests (i.e., squat jump [SJ], countermovement jump [CMJ], standing long jump [SLJ], and 3-hop jump [3HJ]), linear sprint tests (i.e., 5 and 20 m dash), and dynamic balance tests (i.e., Y-balance test). Significant correlations ranging from moderate to large [47] between TSAT and SJ (*r* = −0.62), CMJ (*r* = −0.43), SLJ (*r* = −0.60) and 3HJ (*r* = −0.62) led the authors to suggest a positive influence of the vertical component of ground reaction force on sport-specific COD performance. It is important to note that the desired outcome in the TSAT test is a smaller number (owing to time being the outcome measure). As such, a negative relationship indicates that those athletes who jump higher are the ones that are typically faster. In parallel, TSAT showed significant and large [47] correlations with the 5 m sprint (*r* = 0.52), 20 m sprint (*r* = 0.58) and Y-balance test (*r* = −0.59), thus highlighting the relevance of other fitness components to the ability to rapidly execute planned multidirectional technical actions [12].

### 5.2. Taekwondo-Specific Aerobic–Anaerobic-Agility (TAAA) Test

**Figure 3:**
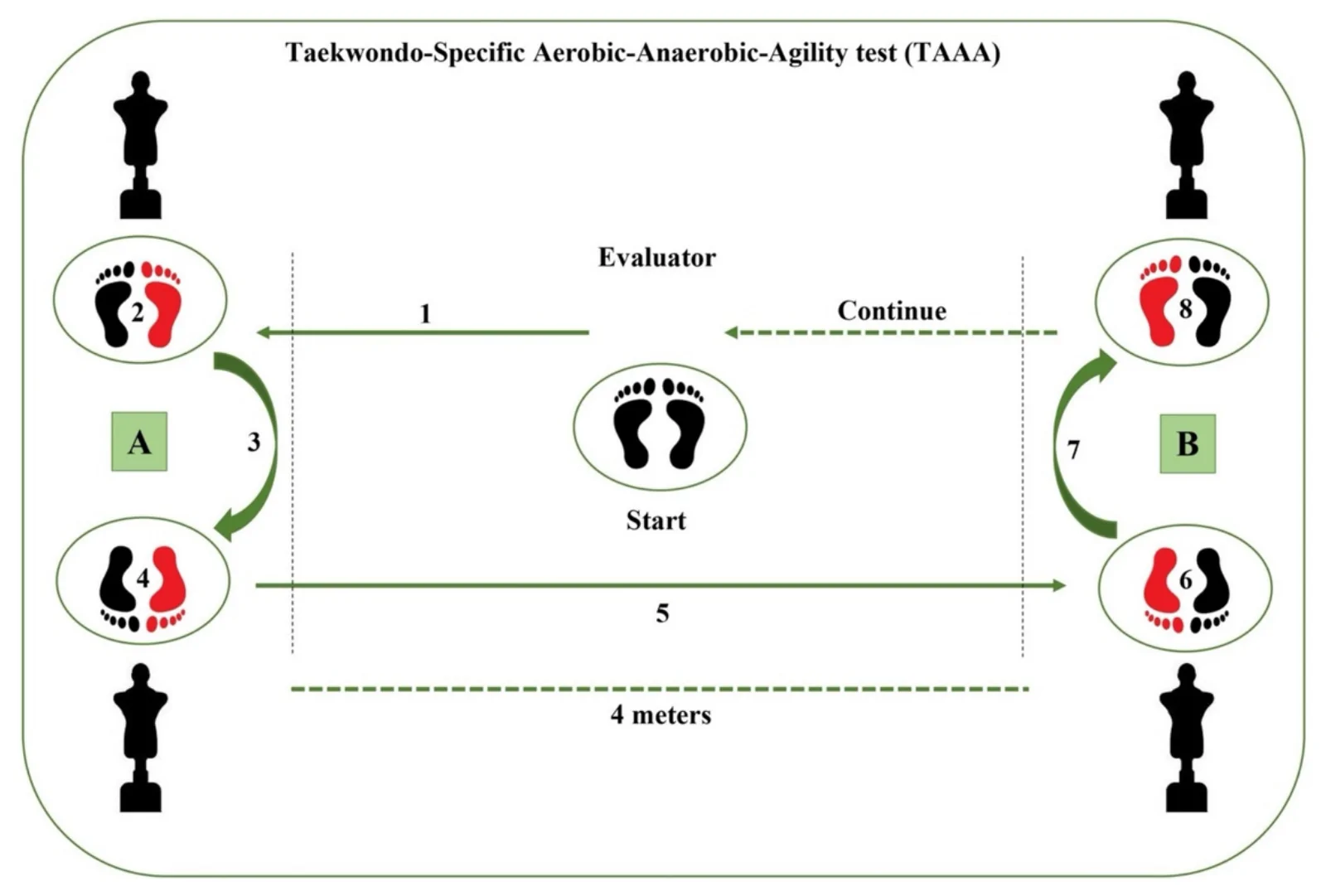
Taekwondo-Specific Aerobic–Anaerobic-Agility (TAAA) test [40]. The test involves six 20 s (a total of 2 min) intervals of shuttle sprints over a 4 m distance, and the execution of the bandal-chagi (i.e., roundhouse kicks) to the punching bags alternating the legs at the end of each distance, with 10 s rest intervals between the sets. The athlete starts the test from a ready position with both heels on the ground and 1 shoulder width between the feet (i.e., naranhi seogi) at the point “start,” in front of the evaluator. After command, the athlete had to: (A) Sprint forwards to point A and perform a right-foot kick to the punching bag 1 from a fighting stance, a right-side 180-degree rotation and a left-foot kick to the punching bag 2 from a fighting stance, (B) sprint forwards to point B and perform the same procedure as those at point A to the punching bags 3 and 4. Prior to the test, each athlete performs several bandal-chagui with a typical scoring impact to establish a reference kicking impact for subsequent visual inspection by evaluator. Each kick is deemed correct when the following criteria are achieved: performing behind the lines defining the 4-m distance on both sides; kicking with the instep of the foot at a height between the navel and nipples; kicking with a typical scoring impact. The time needed to perform six correct kicks is recorded and represents COD performance. A standard stopwatch is used to record time measurement in seconds. The shortest time from two attempts with a 3 min recovery period between them is considered for subsequent analysis.

Taati et al. [40] developed the TAAA test, which consists of performing shuttle sprints and six kicks (i.e., bandal-chagi) over a distance of 4 m, using four punching bags. COD performance is determined by executing the test in the shortest possible time, using an overall technique deemed effective by the evaluator (Figure 3).

The authors’ main objective was to investigate the concurrent criterion validity of the TAAA test with the *t*-Test. The average CV values for the TAAA test and the *t*-Test were 6.9% and 7%, respectively. A significant and very large [47] correlation (*r* = 0.73) and a 53% shared variance between the performance of the two tests were found [40]. In parallel, Taati et al. [40] investigated the discriminant construct validity of the TAAA test by comparing performance between regional- and national-level athletes. The ROC curve analysis revealed an area under the curve of 0.82 for COD performance, suggesting a high ability of the TAAA test to discriminate between these two groups of athletes [40]. Taati et al. [40] found a COD performance of 12.1 ± 0.8 s in the TAAA test when testing 50 Iranian males. In addition, they developed a classification table (≥13.5 s: poor; 12.8–13.4 s: regular; 11.6–12.7 s: good; ≤11.5 s: excellent) as a tool for easily monitoring and classifying athletes’ performance. In this regard, it is worth noting that classification thresholds derived from relatively small samples should be interpreted as study-specific values rather than universal normative standards.

### 5.3. Taekwondo-Specific Change-of-Direction Speed Test (TST)

**Figure 4:**
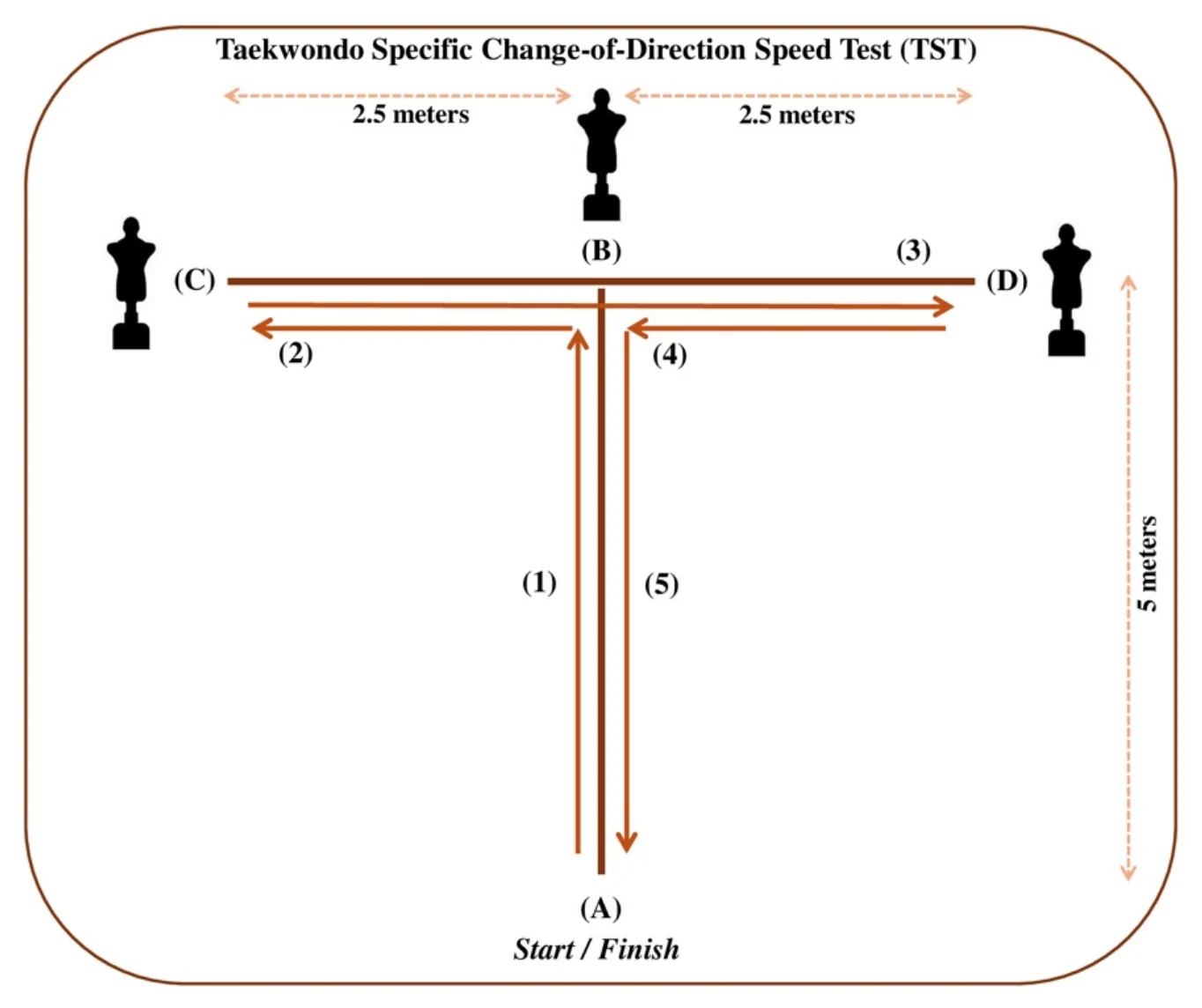
Taekwondo-Specific Change-of-Direction Speed Test (TST) [27]. From a guard position with both feet behind the start/finish line (A), the athlete has to: (1) move forward (left leg forward) towards the dummy (B), in guard position, with chasing steps and without crossing feet, and perform a right direct punch on an electronic body protector; (2) turn and move towards the dummy (C) with a lateral shift with chasing steps (left leg forward) and perform a yop-chagi (i.e., side kick) with the left leg, followed by a bandal-chagi (i.e., roundhouse kick) with the right leg on an electronic body protector; (3) change direction, turn and move towards the dummy (D) by adopting a lateral shift with chasing steps (right leg forward) and perform a yop-chagi with the right leg, followed by a bandal-chagi with the left leg on an electronic body protector; (4) change direction, turn and return to the center, moving towards the dummy (B) by adopting a lateral shift with chasing steps (left leg forward), and perform a bandal-chagi with a leg of the athlete’s choice against an electronic headguard protector, followed by a dwit-chagi (i.e., spin back kick) with the other leg against an electronic body protector; and (5) move back to the start/finish line (A) in a guard position, by adopting a lateral shift with chasing steps and without crossing feet. Athlete is asked to wear his/her official protectors during the test. The height of the dummy is adjusted to the same height as the athlete. If the athlete crosses their feet during the lateral shift with chasing steps or falls to the ground during the execution of the different kicks, the trial is invalidated and a new attempt is granted after a 3 min recovery period. The athlete performs three trials, and the best one is used for subsequent analysis. The TST performance variables are the time needed to complete the test (TST_TO_), assessed with an electronic timing system; the time needed for striking penalty (TST_TSP_) (i.e., the sum of the TST_TO_ and the number of non-scored strikes multiplied by 0.5 s); the time required to complete striking techniques (TECHN_INDEX_) (i.e., the difference between the TST_TO_ and the modified *t*-Test score).

Aloui et al. [27] developed the TST, which consists of performing specific movements, one punch and six kicks (i.e., yop-chagi, bandal-chagi, and dwit-chagi), within a 5 m × 5 m area, using three dummies fitted with electronic body and/or headguard protectors. TST performance is determined not only by the time needed to complete the test (TST_TO_), but also by the time needed with striking penalty (TST_TSP_) and the time required to complete striking techniques (TECHN_INDEX_), with technical execution judged effective by the evaluator (for movements and punches) and by electronic devices (for kicks) (Figure 4).

In line with Chaabene et al. [12], the authors simultaneously investigated certain aspects of the validity, reliability, and resulting sensitivity of the TST [27]. Specifically, the concurrent criterion validity of the new test was assessed with the modified *t*-Test. Large [47] (*r* = 0.69) and very large [47] (*r* = 0.71) significant correlations between TST performance (TST_TO_ and TST_TSP_, respectively) and that of the general test were observed. Furthermore, stratifying the analyses by sex, the magnitude of the correlations was confirmed for both males (TST_TO_: *r* = 0.57; TST_TSP_: *r* = 0.72) and females (TST_TO_: *r* = 0.60; TST_TSP_: *r* = 0.59) [27]. On the other hand, the discriminant construct validity of the TST was examined by comparing the performance of national- and international-level athletes. International athletes showed significantly (ES = 1.6, 2.0, and 1.2, respectively; “large”) higher TST_TO_, TST_TSP_ and TECHN_INDEX_ performance (8.9 ± 0.7 s, 10.3 ± 0.6 s, and 2.1 ± 0.4 s, respectively) than their national counterparts (10.2 ± 0.9 s, 11.8 ± 0.9 s, and 2.9 ± 0.8 s, respectively). In this regard, the ROC curve analysis showed an area under the curve of 0.88 (with a cutoff value of ≤9.54 s) for the TST_TO_ and 0.99 (with a cutoff value of ≤10.78 s) for the TST_TSP_; thus, this demonstrates a high discriminatory capacity of the TST [27]. Furthermore, additional comparisons by sex and competitive level revealed significantly (ES = 0.6, 1.9, and 1.7, respectively; “small” to “large”) higher TST_TO_, TST_TSP_ and TECHN_INDEX_ performance in international female athletes (9.3 ± 0.4 s, 10.6 ± 0.3 s, and 2.3 ± 0.1 s, respectively) compared to national male athletes (9.6 ± 0.4 s, 11.1 ± 0.1 s, and 2.9 ± 0.5 s, respectively). In the validation study, Aloui et al. [27] tested 10 Tunisian males (TST_TO_: 8.9 ± 1.0 s; TST_TSP_: 10.6 ± 1.1 s) and 10 Tunisian females (TST_TO_: 10.1 ± 0.9 s; TST_TSP_: 11.7 ± 1.3 s).

Regarding the test–retest reliability analysis, the ICC (males: TST_TO_ = 0.86, TST_TSP_ = 0.80; females: TST_TO_ = 0.92, TST_TSP_ = 0.80) and CV (males: TST_TO_ = 4.4%, TST_TSP_ = 4.6%; females: TST_TO_ = 2.9%, TST_TSP_ = 5.4%) established high relative and absolute reliability [18,26] for both males and females [27]. However, the sensitivity analysis revealed that the TST is not suitable for detecting small but meaningful changes in performance due to its lower SWC_0.2_ values (males: TST_TO_: 1.9 s, TST_TSP_: 1.6 s; females: TST_TO_: 1.7 s, TST_TSP_: 1.8 s) compared to the TEM (males: TST_TO_: 4.4 s, TST_TSP_: 4.6 s; females: TST_TO_: 2.9 s, TST_TSP_: 5.4 s), in both sexes [27].

The secondary objective of the study by Aloui et al. [27] was to determine the relationship of the TST with lower-limb muscle power tests (SJ, CMJ, single-leg hop [SLH], single-leg triple hop [SLTH], and 5-jump [5-J]), linear sprint tests (i.e., 5, 10, 20, and 30 m), and dynamic balance tests (i.e., standing stork balance and Y-balance test). Moderate-to-large [47] correlations were found between TST performance and lower-limb muscle power performance in males (TST_TO_: *r* = −0.34–−0.53; TST_TSP_: *r* = −0.65–−0.45) and low-to-nearly perfect [47] correlations in females (TST_TO_: *r* = −0.92–0.42; TST_TSP_: *r* = −0.87–0.31). Notably, the authors emphasized the high correlation between the sport-specific COD test and the single-leg jump test, as the TST involves decelerations and re-accelerations during changes in direction and unilateral techniques [27]. In parallel, correlation analyses between TST performance and linear sprint performance revealed low-to-large [47] relationships for males (TST_TO_: *r* = 0.17–0.34; TST_TSP_: *r* = 0.27–0.51) and large-to-very large [47] relationships for females (TST_TO_: *r* = 0.54–0.85; TST_TSP_: *r* = 0.62–0.81). Overall, these results suggest that the magnitude of the correlation might depend on a certain degree of similarity between the sport-specific COD test and the specific distance of the linear sprint [27]. In addition, moderate-to-very large [47] correlations were found between TST performance and dynamic balance test performance in both males (TST_TO_: *r* = −0.64–−0.52; TST_TSP_: *r* = −0.74–−0.42) and females (TST_TO_: *r* = −0.71–−0.44; TST_TSP_: *r* = −0.55–−0.39), as athletes require good balance (both static and dynamic) when performing particularly complex technical actions [27].

## 6. Discussion

The presented testing protocols (i.e., TSAT, TAAA test, and TST) allow for monitoring COD ability through an ecological approach and support the work of scientists, coaches, and strength and conditioning professionals due to their simplicity and non-invasive nature. Ecological validity indicates the extent to which a test reflects an athlete’s actual sporting performance and real sporting context [68]. Therefore, this aspect of validity is the basis for sport-specific assessment and is common to the three tests currently available. On the other hand, it is relevant to highlight that the ecological validity of each test was based on the scientific literature and/or on an attempt to improve upon a previous protocol; thus, this determines distinct methodological and measurement characteristics that warrant discussion.

### 6.1. Methodological and Measurement Characteristics

The rapid whole-body movements, accompanied by changes in speed and direction, typical of the TSAT are performed in a guard stance and without crossing the feet, to mimic the position maintained during the low-intensity active phases of the match. In contrast, the kick techniques (i.e., dollyo-chagi and narae-chagi), which occur between the movements in this test, were chosen as they are commonly performed during a match. Furthermore, the test is performed in a 4 m × 4 m area, as the athletes typically interact at close range within a confined area [12]. Although these methodological characteristics support the ecological validity of the TSAT, Chaabene et al. [12] have suggested that future studies could develop a modified version of the test that includes decision-making tasks to assess agility. The TSAT is the first test developed to assess COD ability using a sport-specific approach and is also the most widely used test in practice. Most studies have focused on investigating the acute effects of different training regimens or conditioning activities and the use of ergogenic supplements, music, or a combination of these on TSAT COD performance [49,56,57,58,59,60,61,62,63,66]. In parallel, other studies have investigated the long-term effects of different training programs, lasting between 1 and 8 weeks, on TSAT COD performance [28,34,44,51,55,64,67]. Instead, only a limited number of studies have established the relationship between TSAT COD performance and that of other key fitness components [50,52,53,54,65] or have compared athletes’ performance in this sport-specific test [41].

In contrast to the TSAT, the typical movements in the TAAA test consist of non-standardized shuttle sprints, which future studies may replace with more specific movements. The distance between each shuttle is 8 m as the octagonal competition area has a diameter of 8 m. Furthermore, the choice of six kicks in this test was based on the typical duration of the *t*-Test, while the most commonly used kicking technique (i.e., bandal-chagi) during the match was performed between the shuttle runs [40]. The TST involves lateral movements forwards and backwards within a 5 m × 5 m area to mimic the position maintained during the low-intensity active phases of a match, which replace the general movements characteristic of the modified *t*-Test. To improve upon the TSAT protocol, the different kicking techniques (i.e., yop-chagi, bandal-chagi, and dwit-chagi) and punches included in the TST were selected to simulate the wide variety of actions performed by athletes in the most recent competitions following rule changes. In addition, Aloui et al. [27] included TST_TSP_ (i.e., the time needed with striking penalty) and TECHN_INDEX_ (i.e., the time required to complete striking techniques) among the TST parameters, as the performance of the TSAT (and the TAAA test) is determined solely by the time needed to complete the test. In line with the suggestion to develop a further version of the TSAT that includes decision-making to simulate a dynamic situation [12], Aloui et al. [27] supported the idea that the TST should also be appropriately modified in the future to combine changes of direction with cognitive measures. The TAAA test and the TST were developed in parallel, and subsequently to the TSAT, for the assessment of COD ability using a sport-specific approach, and no study has yet utilized these tests in investigations using different interventions (e.g., training, ergogenic supplements). However, it is worth noting that a table has been provided to classify the performance of the TAAA test, and this should also be developed for the TSAT and TST [40].

### 6.2. Validity

Although ecological validity is the key element of sport-specific tests, all types of validity (i.e., translational, criterion and construct) are important in the assessment of physical qualities, and it is also necessary to provide evidence for each of their different subdomains [68]. The content translational validity (i.e., the extent to which the content of a test matches and measures all elements of a given construct [68]) of the TSAT, TAAA test and TST was established through an in-depth analysis of the relevant scientific literature. In contrast, face translational validity (i.e., what a test superficially seems to measure, regardless of what it actually measures [68]) has not been addressed in any test. The concurrent criterion validity (i.e., the strength of the relationship and agreement between the sport-specific test and the established reference COD test [45]) of the TSAT and TAAA test was defined using the *t*-Test, while that of the TST was defined using the modified *t*-Test. This validity subdomain has been established for all available COD ability tests, thus representing one of their main strengths. In contrast, the predictive criterion validity (i.e., the extent to which a sport-specific outcome can predict sport performance [68]) of the TSAT, TAAA test and TST has not been explored. In this regard, the multifactorial nature of taekwondo performance may preclude any single test from accurately predicting performance in this sport [6,7]. The discriminant construct validity (i.e., the ability of a sport-specific test to assess performers of different abilities [45]) of the TSAT, TAAA test and TST was determined by comparing performance among athletes of different competitive levels (i.e., regional vs. national or national vs. international) using a statistical approach based on ROC curve analysis. In this regard, small sample sizes and potential imbalance between male and female participants could limit the study of discriminant validity [6,7]. In this brief review, the number of participants recruited for the studies ranged from 13 to 52, comprising 373 males and 213 females, thereby highlighting a balance in the study of sport-specific COD assessment. Convergent construct validity (i.e., the extent to which a sport-specific test and another measure of the same construct are indeed related [68]) logically represents a subdomain that has yet to be explored, as the TSAT has been the only test available for several years to assess this component of physical fitness in sport-specific settings.

### 6.3. Reliability and Sensitivity

In addition to the evidence from the different subdomains of validity, determining test–retest reliability (i.e., the consistency of measurements or of a performance over repeated testing sessions [45]) is essential to establish with certainty whether an athlete has actually improved performance in a test. The test–retest reliability of the TSAT has been systematically documented, as has its intra-session reliability (as multiple attempts are generally allowed to establish the best COD performance). In contrast, these reliability aspects still represent a major methodological shortcoming for other tests (i.e., the TAAA test and the TST) developed subsequently. On the other hand, intra- and inter-rater reliability (i.e., the degree of agreement between assessment outcomes when undertaken by the same or two testers [45]) has not been established for any of the tests, and this represents a concern, particularly for the TSAT and TAAA test, as their performance is deemed valid only if the test is executed using an overall technique judged effective by the evaluator. This problem has been partially resolved in the TST through the use of electronic body and headguard protectors to objectively measure the impact of kicks. This solution could also be extended to the TST punching technique (given its recent introduction into competition [2]) and should also be implemented in the TSAT and TAAA test. Finally, the sensitivity (i.e., the extent to which a sport-specific test is able to detect small but meaningful changes in performance [45]) of the TSAT and TST was investigated by calculating the SWC and/or MDC_95%_. However, the TST did not show adequate sensitivity, while these analyses were not performed for the TAAA test, thus limiting their practical applicability. In this regard, a statistically reliable test is not necessarily sufficiently sensitive for longitudinal monitoring. Practitioners should, therefore, consider measurement error and minimal detectable/meaningful change when interpreting changes following training.

### 6.4. Limitations

This narrative review is subject to several methodological limitations that warrant consideration. First, a substantial amount of the available literature focuses on TSAT, leading to an over-representation of this test in the body of evidence. The other tests, namely the TAAA test and the TST, remain under-represented, with only studies on their development and validation processes available. This limits the comparison of the tests and their results. Second, sample characteristics may restrict the generalizability of the results. Most of the literature comes from African or South American countries. Furthermore, the different competitive levels and age categories of the recruited athletes add further heterogeneity to the data. Finally, although the authors’ direct experience with the topic under investigation aligns with the methodology of narrative reviews, it represents a potential bias to be considered in the discussion of the results.

## 7. Practical Applications

**Figure 5:**
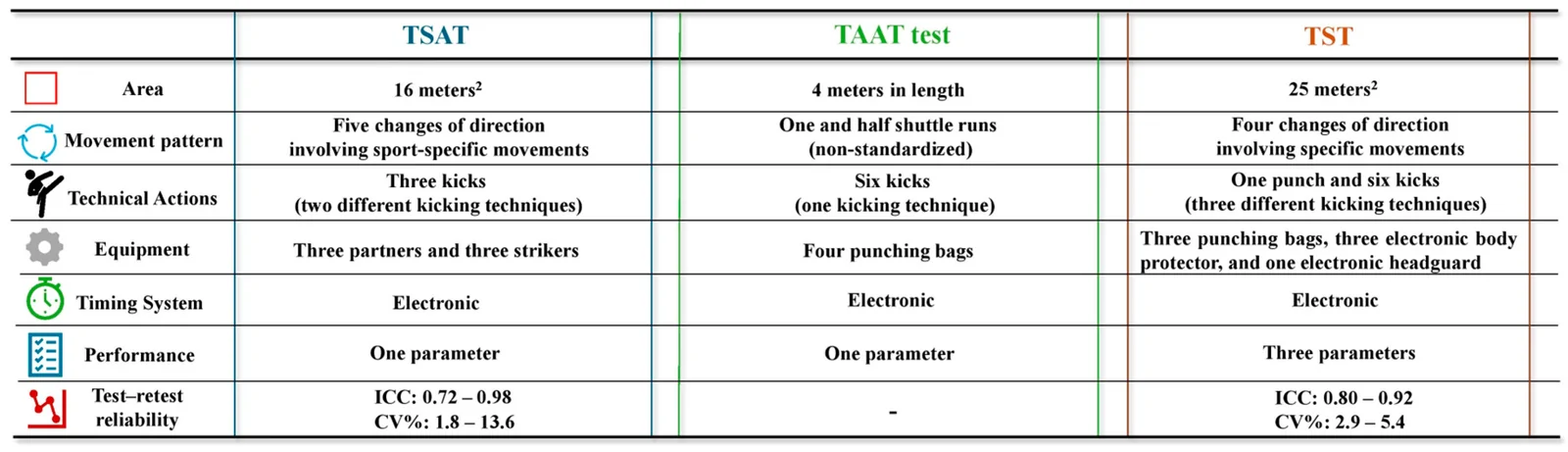
Methodological and measurement characteristics of the Taekwondo-Specific Agility Test (TSAT), Taekwondo-Specific Aerobic–Anaerobic-Agility (TAAA) test, and Taekwondo-Specific Change-of-Direction Speed Test (TST). Notes: ICC = intraclass correlation coefficient (<0.5: poor; 0.5–0.75: moderate; 0.76–0.9: good; >0.9: excellent [47]); CV% = coefficient of variation in percentage (<5%: excellent; <10%: very good; <15%: acceptable; >15%: poor [48]).

The overview provided in this brief review on the sport-specific assessment of COD ability in taekwondo has highlighted the availability of test protocols (i.e., TSAT, TAAA test, and TST) that incorporate greater sport-specificity and may provide greater ecological relevance than general tests (*t*-Test, modified *t*-Test, 10 × 5 m shuttle run test), as they mimic the typical movements and technical actions of the sport. Each test presents distinctive methodological (i.e., area dimensions, movements, kicking and punching techniques, equipment) and measurement (i.e., performance parameters) characteristics that must be considered before practitioners decide which one is most suitable for their athletes and context. In this sense, strength and conditioning professionals can refer to the infographic (Figure 5), which provides an overview of each sport-specific test method, its associated movement demands, equipment requirements, and the typical reliability that practitioners can expect from each. The rapid whole-body movements, accompanied by changes in speed and direction, typical of the TSAT are performed in a 4 m × 4 m area. The dollyo-chagi and narae-chagi that occur between the movements in this test are performed [12]. The typical movements in the TAAA test consist of non-standardized shuttle sprints. The distance between each shuttle is 8 m. The bandal-chagi is performed between the shuttle runs [40]. The TST involves lateral movements forwards and backwards within a 5 m × 5 m area. The yop-chagi, bandal-chagi, dwit-chagi and punches are performed between the movements [27]. In this context, given the equipment required for each test, practitioners are advised to use standardized evaluation criteria and, where possible, objective/electronic scoring procedures, particularly when technical execution determines whether a trial is considered successful. From a scientific perspective, scientists should improve the ecological validity of existing tests by replacing general movements (such as shuttle runs), using techniques that reflect changes in the technical–tactical profile of current matches, incorporating performance parameters that include the technical component, and developing updated versions that take cognitive measures into account for assessing agility. In fact, the sport-specific tests may themselves require periodic revision as competition rules and technical–tactical behaviors evolve. Furthermore, they should explore further aspects of validity (such as face, convergent and predictive validity), clarify the reliability (i.e., test–retest and/or intra-/inter-rater) and sensitivity of these tests, and develop classification tables. In particular, although a test reproduces sport-specific movements, it does not demonstrate that it predicts actual competitive performance. Future studies should examine whether performance in these tests is related to meaningful competitive outcomes or technical–tactical performance indicators. In addition, the examination of the concepts of reliability and sensitivity will assist coaches and strength and conditioning professionals in using tests to monitor the acute and chronic effects of training protocols on sport-specific COD performance, to monitor the relationship between the latter and other key components of fitness, or to classify athletes.

## Figures and Tables

**Table 1:** Performance variable measured during the execution of general change of direction (COD) tests in taekwondo athletes.

Study	Athlete Characteristics (n°, Sex, Country)	Test	Time (s)
Singh et al. [29]	National (30 Indian males)	Illinois Agility Test	17.1 ± 0.4 a 17.1 ± 0.4 ab
Arazi et al. [36]	Regional and national (40 Iranian females)	4 × 9 m shuttle run test	11.6 ± 1.6
Nikolaidis et al. [32]	NR (Greek, 221 males, 172 females) • 7–9 years (males) • 10–11 years (males) • 12–13 years (males) • 14–17 years (males) • 18–32 years (males) • 33+ years (males) • 7–9 years (females) • 10–11 years (females) • 12–13 years (females) • 14–17 years (females) • 18–32 years (females) • 33+ years (females)	10 × 5 m shuttle run test	19.8 ± 1.7 18.6 ± 1.3 18.0 ± 1.5 16.1 ± 1.0 15.8 ± 1.1 16.9 ± 1.7 20.1 ± 1.7 19.3 ± 1.3 18.4 ± 1.1 17.8 ± 1.3 17.2 ± 1.4 20.4 ± 1.6
Herrera-Valenzuela et al. [31]	NR (Chilean, 11 males, 11 females)	10 × 5 m shuttle run test	21.9 ± 1.2 a
Monks et al. [26]	National (Korean, 17 males, 16 females)	*t* -Test	11.1 ± 0.1 a 11.4 ± 0.1 a
Singh et al. [30]	National (30 Indian males)	Illinois Agility Test	17.8 ± 1.9 a 18.9 ± 2.2 ab
Chaabene et al. [12]	National and international (Tunisian, 20 males, 7 females)	*t* -Test	9.9 ± 0.6
Jung et al. [37]	NR (Korean, 21 males, 14 females)	*t* -Test	10.4 ± 0.1 a 10.3 ± 0.1 ac
Seo et al. [38]	National (47 Korean males)	*t* -Test	10.5 ± 0.3 a 10.4 ± 0.2 a 10.6 ± 0.2 a 10.3 ± 0.6 ab
Seo et al. [39]	National (47 Korean males)	*t* -Test	10.5 ± 0.5 10.5 ± 0.4 10.5 ± 0.3
Ouergui et al. [28]	Regional and national (Tunisian, 27 males, 9 females)	Modified *t*-Test	6.5 ± 0.5 a 6.8 ± 0.6 a 7.2 ± 0.7 ab
Taskin and Akkoyunlu et al. [35]	National (16 Turkish males)	*t* -Test	9.8 ± 0.4
Chen et al. [33]	National (15 Taiwanese males)	5–0–5 Agility Test Hexagon Agility Test	2.6 ± 0.2 b 12.8 ± 1.6 b
Taati et al. [40]	Regional and national (50 Iranian males)	*t* -Test	11.1 ± 0.8
Aloui et al. [27]	National and international (Tunisian, 10 males, 10 females) • Males • Females	Modified *t*-Test	6.7 ± 0.4 7.6 ± 0.4
Avci and Celik [41]	National and international (Turkish, 14 males, 13 females) • 11–12 years • 13–15 years	Zig-zag Agility Test	7.3 ± 0.4 6.8 ± 0.4
Khazaei et al. [42]	National and international (17 Iranian females)	*t* -Test	11.7 ± 0.7 a 11.6 ± 0.6 a
Shen [34]	National and international (30 Chinese females)	5–0–5 Agility Test	2.7 ± 0.2 a 2.6 ± 0.2 a
Laurin [43]	NR (American, 265 males, 205 females)	Pro Agility (5–10–5) Test	5.2 ± 0.4
Huang et al. [44]	NR (Chinese, 14 males, 16 females)	Hexagon Agility Test	13.7 ± 1.7 a 13.2 ± 1.4 a
Hsieh et al. [25]	Regional and national (Taiwanese, 16 males, 12 females)	*t* -Test	12.5 ± 1.0

Notes: a = pre-intervention values; b = control condition values; c = placebo condition values; NR = Not reported. Values expressed as mean ± SD.

**Table 2:** Test–retest reliability, intra-session reliability, and performance variable measured during the execution of the Taekwondo-Specific Agility Test (TSAT) in taekwondo athletes.

Study	Athlete Characteristics (n°, Sex, Country)	Test–Retest Reliability	Intra-Session Reliability (n° Trials)	Time (s)
Chaabene et al. [12]	National and international (Tunisian, 20 males, 7 females)	ICC: 0.97 CV%: 1.8	NR (three)	5.3 ± 0.6
Ojeda-Aravena et al. [53]	Regional and national (14 Chilean males and females)	NR	NR (three)	7.8 ± 0.9
Ouergui et al. [28]	Regional and national (Tunisian, 27 males, 9 females)	ICC: 0.86	NR (three)	6.7 ± 0.5 a 7.1 ± 0.7 a 7.0 ± 0.4 ab
Ojeda-Aravena et al. [54]	Regional and national (14 Chilean males and females)	NR	NR (three)	7.8 ± 0.9
Ojeda-Aravena et al. [51]	National (Chilean, 11 males, 5 females)	NR	ICC: 0.90 a ICC: 0.86 d (two)	8.0 ± 1.1 a 7.8 ± 1.0 ab
Ouergui et al. [55]	Regional and national (32 Tunisian males)	ICC: 0.82	NR (three)	6.7 ± 0.1 a 7.0 ± 0.4 a 6.8 ± 0.2 a 7.2 ± 0.5 ab
Ouergui et al. [49]	Regional and national (Tunisian, 10 males, 10 females)	ICC: 0.98	NR (three)	6.2 ± 0.3 b
Ouergui et al. [56]	Regional and national (Tunisian, 14 males, 13 females)	ICC: 0.85	NR (three)	7.0 ± 0.9 b
Avci and Celik [41]	National and international (Turkish, 14 males, 13 females) • 11–12 years • 13–15 years	NR	NR (two)	5.9 ± 0.3 5.4 ± 0.4
Ouergui et al. [57]	Regional and national (Tunisian, 10 males, 10 females)	ICC: 0.85	NR (three)	6.3 ± 0.4 b
Ouergui et al. [58]	Regional and national (Tunisian, 10 males, 10 females)	ICC: 0.86	NR (three)	NR b
Ouergui et al. [59]	Regional and national (Tunisian, 13 males, 8 females)	ICC: 0.93	NR (three)	RF b
Ouergui et al. [60]	Regional, national and international (Tunisian, 26 males, 26 females) • Elite (16 males, 16 females) • Sub-elite (10 males, 10 females) • Elite (16 males, 16 females) • Sub-elite (10 males, 10 females)	ICC: 0.86	NR (three)	5.9 ± 0.4 b 6.2 ± 0.4 b 5.7 ± 0.3 c 6.1 ± 0.7 c
Delleli et al. [61]	National and international (16 Tunisian males)	ICC: 0.88	NR (three)	5.7 ± 0.3 b
Ojeda-Aravena et al. [50]	National and international (Chilean, 8 males, 9 females)	NR	ICC: 0.80 CV%: 10 (three)	6.7 ± 0.9
Messaoudi et al. [62]	National (16 Tunisian males)	ICC: 0.95	NR (three)	6.4 ± 0.3 b
Shen [34]	National and international (30 Chinese females)	NR	ICC: 0.90 a ICC: 0.86 d (two)	8.0 ± 0.4 a 8.3 ± 0.4 a
Delleli et al. [63]	National and international (16 Tunisian females)	ICC: 0.87	NR (three)	6.2 ± 0.3 b
Huang et al. [44]	NR (Chinese, 14 males, 16 females)	ICC: 0.72 CV%: 13.6	NR (three)	8.7 ± 1.2 a 8.3 ± 0.9 a
Song and Sheykhlouvand [64]	Regional (30 Iranian males)	NR	NR (NR)	5.6 ± 0.4 a 5.5 ± 0.6 a 5.5 ± 0.5 ab
Samanipour et al. [65]	National and international (Iranian, 20 males, 15 females) • Males • Females	NR	NR (three)	8.0 ± 0.6 6.1 ± 0.8
Nana et al. [66]	Regional and national (13 Thais males)	NR	NR (two)	5.4 ± 0.5 ac 5.7 ± 0.8 a
Ojeda-Aravena et al. [52]	National and international (Chilean, 9 males, 6 females)	NR	ICC: 0.84 CV%: 9.3 (NR)	6.9 ± 0.9
Herrera-Valenzuela et al. [67]	NR (17 Chilean males and females) • Males • Females	NR	NR (NR)	6.6 ± 0.4 a 7.5 ± 0.6 a

Notes: ICC = intraclass correlation coefficient (<0.5: poor; 0.5–0.75: moderate; 0.76–0.9: good; >0.9: excellent [47]); CV% = coefficient of variation in percentage (<5%: excellent; <10%: very good; <15%: acceptable; >15%: poor [48]); NR = not reported; a = pre-intervention values; b = control condition values; c = placebo condition values; d = post-intervention values, RF = reported in figure (see the reference). Values expressed as mean ± SD.

## Data Availability

No new data were created or analyzed in this study. Data sharing is not applicable to this article.

## Abbreviations

The following abbreviations are used in this manuscript:

COD	Change of direction
OG	Olympic Games
TSAT	Taekwondo-Specific Agility Test
SANRA	Scale for the Assessment of Narrative Review Articles
OSF	Open Science Framework
TAAA	Taekwondo-Specific Aerobic–Anaerobic-Agility test
TST	Taekwondo-Specific Change-of-Direction Speed Test
ES	Effect size
ROC	Receiver operating characteristic
ICC	Intraclass correlation coefficient
CV	Coefficient of variation
SWC	Smallest worthwhile change
TEM	Typical error of measurement
MDC	Minimal detectable change
SJ	Squat jump
CMJ	Countermovement jump
SLJ	Standing long jump
3HJ	3-hop jump
TST_TO_	Time needed to complete the test
TST_TSP_	Time needed with striking penalty
TECHN_INDEX_	Time required to complete striking techniques
SLH	Single-leg hop
SLTH	Single-leg triple hop
5-J	5-jump
